# Capacity changes in German certified chest pain units during COVID-19 outbreak response

**DOI:** 10.1007/s00392-020-01676-z

**Published:** 2020-05-31

**Authors:** Stephan Settelmeier, Tienush Rassaf, Evangelos Giannitsis, Thomas Münzel, Frank Breuckmann

**Affiliations:** 1 Department of Cardiology and Vascular Medicine, West German Heart and Vascular Center Essen, University Duisburg-Essen, Essen, Germany; 2 3rd Department of Medicine, University Hospital Heidelberg, Heidelberg, Germany; 3 Department of Cardiology, University Medical Center Mainz, Johannes Gutenberg-University Mainz, Mainz, Germany

**Keywords:** Chest pain unit, Germany, Capacity, COVID-19, Pandemic

## Abstract

**Background:**

We sought to determine structure and changes in organisation and bed capacities of certified German chest pain units (CPU) in response to the emergency plan set-up as a response to the SARS-CoV-2 pandemic.

**Methods and results:**

The study was conducted in the form of a standardised telephone interview survey in certified German CPUs. Analyses comprised the overall setting of the CPU, bed capacities, possibilities for ventilation, possible changes in organisation and resources, chest pain patient admittance, overall availability of CPUs and bail-out strategies. The response rate was 91%. Nationwide, CPU bed capacities decreased by 3% in the early phase of COVID-19 pandemic response, exhibiting differences within and between the federal states. Pre-pandemic and pandemic bed capacities stayed below 1 CPU bed per 50,000 inhabitants. 97% of CPUs were affected by internal reorganisation pandemic plans at variable extent. While we observed a decrease of CPU beds within an emergency room (ER) set-up and on intermediate care units (ICU), beds in units being separated from ER and ICU were even increased in numbers.

**Conclusions:**

Certified German CPUs are able to maintain adequate coverage for chest pain patients in COVID-19 pandemic despite structural changes. However, at this time, it appears important to add operating procedures during pandemic outbreaks to the certification criteria of forthcoming guidelines either at the individual CPU level or more centrally steered by the German Cardiac Society or the European Society of Cardiology.

## Introduction

In Germany, new national regulations urged health care providers to increase capacities for SARS-CoV-2-positive individuals. Hospitals were advised to postpone elective operations/interventions to create free capacities for primary care and to double the capacity of 28,000 intensive care unit (ICU) beds [1–3]. Simultaneously, the German Cardiac Society (GCS) addressed the need for maintenance of guideline-adherent clinical practice in cardiac patients despite the emergency plans for COVID-19 patients [4, 5].

At the time of outbreak, German certified chest pain units (CPUs) are building a nationwide network with currently 287 certified units (due date: March 2020), thereby offering a minimum of four fully monitored beds per unit [6–8]. From a survey from 2016, we know that about half of all CPUs are structured as an integral component of the emergency room (ER) and as much as 11% are even located on an ICU, whereas only about 40% are operated as an independent unit or as part of a cardiologic intermediate care (IMC) unit (40%) [9]. Thus, we hypothesized that the increase of ICU bed capacities as well as the set-off of isolation zones in the ERs might interfere with the given CPU structures. The current study aimed to analyse potential changes of CPU set-up and/or bed capacity based on the individual implementations to the COVID-19 emergency plan by each individual hospital housing a certified CPU.

## Methods

The study design comprised of a standardised telephone interview survey with a questionnaire focusing on the characterisation of the respective CPU. The interview was carried out either by interviewing the head of the CPU or contacting a CPU hotline where available. Following formal oral consent to participate, the CPU’s medical professionals were assisted to respond to the questionnaire. Data collection included all certified CPUs across Germany. Certified units were identified by the official website of the GCS [1]. Telephone interviews were performed within 15 days. The due date was April 1st 2020. At this time, 67,366 individuals were ‘tested positive’, 732 were reported ‘dead’ and 18,700 cases were reported ‘recovered’ in Germany [10].

### Basic CPU characterisation

The CPU characterisation criteria encompassed i) the CPU integration within the hospital facilities, ii) the number of CPU beds per facility, iii) the type of hospital and iv) the capability for mechanical ventilation in the CPU. Thereby, the CPU integration was distinguished into i) CPU as an independent department and/or as an integral part of IMC unit both led by the department of cardiology, ii) CPU as part of the ER or iii) as a part of the ICU. The number of beds was defined as the number of monitored beds fulfilling the CPU criteria according to the GCS. The type of hospital distinguished between i) university hospital, ii) academic teaching hospital and iii) other health facilities provider such as primary care in a community hospital.

### COVID-19 emergency plan CPU reorganisation

Reorganizational characterisation criteria comprised changes in organisation, changes in location, changes in bed capacities and changes in ventilator access for invasive and non-invasive ventilation. In case of any changes, the initiator for the changes was asked, thereby distinguishing between administration, CPU head and health department. Additionally, it was evaluated if the individual CPU is still capable to admit patients. This included evaluation of overall availability, possible bail-out strategies and handling of chest pain patients suggestive for SARS-CoV-2 infection. For the latter, due to possible coincidence and co-dependency of patients presenting with fever, respiratory syndromes and chest pain, we specifically asked where these patients were admitted.

### Demographic characteristics

Data on population were retrieved from the Registry of the Federal Statistical Office (Statistisches Bundesamt) [11].

### Statistics

All data are provided in a descriptive approach without further statistical analysis.

## Results

The response rate to the telephone interview was 91%, allowing data collection from 261 certified units by April 1st 2020.

### Pre-pandemic structure and capacity

Before activation of the national emergency plans for German hospitals, 44% of CPUs were integral part of the ER, 29% were separate units within the department of cardiology, 18% were part of IMC units, while 9% were part of ICUs. A total of 1.738 CPU beds were provided, which equals one CPU bed per 47.697 inhabitants nationwide with differences between the federal states (Table 1). As referred to the internal structural distribution of CPUs, 684 beds were found in ER settings, 568 beds in separate CPUs, 326 beds within IMC units and 156 beds within ICUs. Regarding the type of hospital, most CPUs were affiliated to academic teaching hospitals (194 CPUs), followed by university hospitals (35 CPUs) and other providers of primary health care (32 CPUs).

**Table 1:** Total number of CPUs, demographic data, CPU coverage in beds per inhabitants (pre-/pandemic) and percentual changes per federal state and nationwide

Federal State	CPUs total	CPUs included	Population total	Inhabitants/CPU bed pre-pandemic	Inhabitants/CPU bed pandemic	Change (%)
North Rhine-Westphalia	66	63	17.932.651	42.901	45.864	− 6.5
Bavaria	54	49	13.076.721	47.379	45.723	+ 3.6
Baden-Wuerttemberg	33	30	11.069.533	57.059	57.956	− 1.5
Hesse	24	20	6.265.809	50.126	45.404	+ 10.4
Lower Saxony	27	24	7.982.448	45.099	52.516	− 14.1
Berlin	13	11	3.644.826	65.086	68.770	− 5.4
Rhineland-Palatinate	16	16	4.084.844	34.913	30.946	+ 12.8
Saxony	15	14	4.077.937	49.731	48.547	+ 2.4
Schleswig–Holstein	8	7	2.896.712	61.632	57.934	+ 6.4
Hamburg	8	7	1.841.179	51.144	70.815	− 27.8
Brandenburg	8	7	2.511.917	69.775	69.775	− 22.2
Mecklenburg-West Pomerania	5	5	1.609.675	29.809	33.535	− 11.1
Thuringia	4	4	2.134.393	50.819	50.819	± 0
Saxony-Anhalt	3	2	2.208.321	138.020	184.027	− 25
Saarland	2	2	990.509	30.953	30.953	± 0
Bremen	2	1	569.352	18.978	23.723	− 20
Nationwide	287	261	82.896.827	47.697	49.080	− 2.8

*CPU* chest pain unit

### Changes in structure and capacity during COVID-19 pandemic

**Fig. 1:**
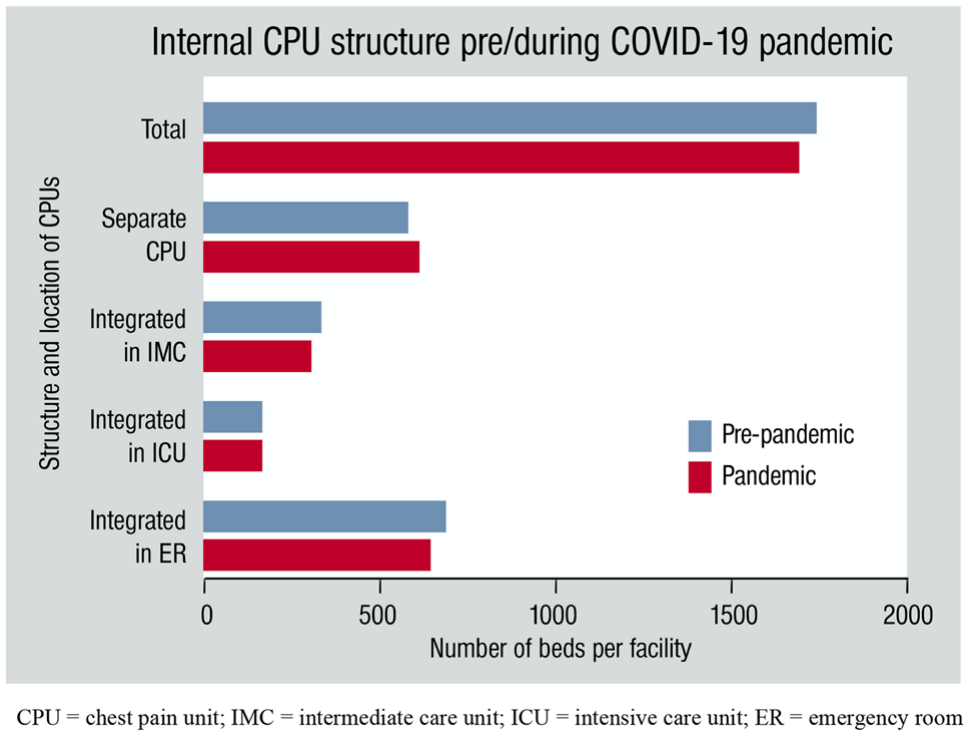
Internal CPU structure pre/during COVID-19 pandemic and resulting amounts of beds. The number of beds per facility is shown according to the structure and location of CPUs. Despite all restructuration during the pandemic, the German certified CPUs were still able to provide a mean of 1 CPU bed per 49.000 inhabitants, which is even higher than in 2016 and still below the goal of more than 1 bed per 50.000 inhabitants as advised by the GCS

Of 261, 97% CPUs stated that they were affected by overall changes due to federal and local pandemic plans to some extent. We observed structural redistribution of CPUs as well as modifications in bed capacities within facilities as a result of preparation for predicted rising numbers of COVID-19 patients. During pandemic, 42% of CPUs were integral part of the ER, 30% were separate units within the department of cardiology, 16% were part of IMC units and 10% were part of ICUs. Eight CPUs (3%) have lost their original CPU structure as functional units. The CPUs now provided 1689 (− 3%) beds in 253 hospitals (− 3%), now offering one bed per 49.080 inhabitants. Of those, 635 (− 7%) were beds in the ER, 610 (+ 7%) in separate CPUs, 288 (− 12%) in IMC units and 156 (± 0%) in ICUs (Fig. 1). While university hospitals increased CPU beds by 4% (+ 10 beds), academic teaching hospitals reduced CPU beds by 3% (− 37 beds) and other providers of primary health care by 9% (− 18 beds) compared to pre-pandemic CPU beds.

### Regional distribution of changes in capacity during COVID-19 pandemic

**Fig. 2:**
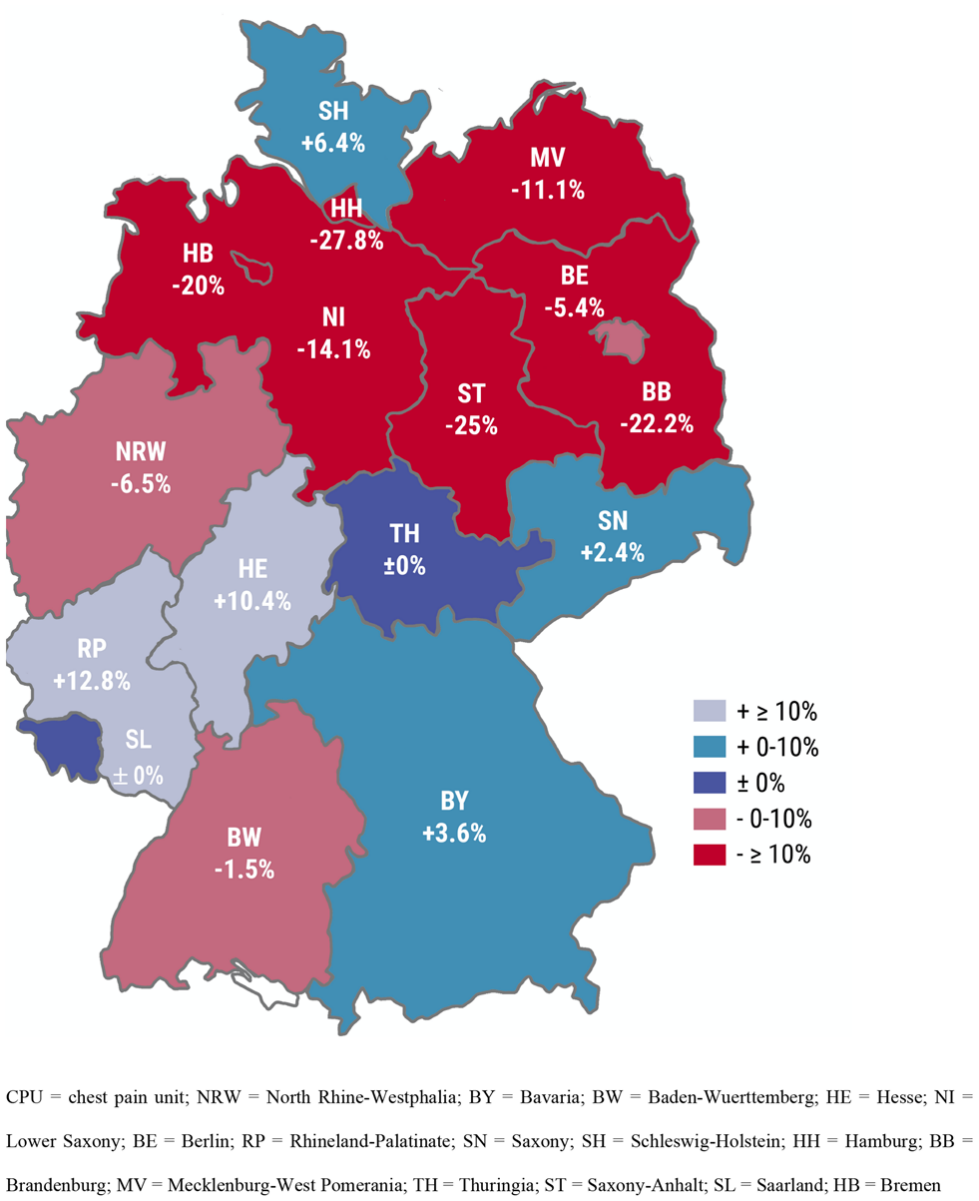
Local changes in CPU beds per inhabitants (federal states of Germany). The distribution of changes in CPU beds per inhabitants showed a north–south gradient with the highest increase in RP and HE and the highest decrease in the states HH and ST. *CPU* chest pain unit, *NRW* North Rhine-Westphalia, *BY* Bavaria, *BW* Baden-Wuerttemberg, *HE* Hesse, *NI* Lower Saxony, *BE* Berlin, *RP* Rhineland-Palatinate, *SN* Saxony, *SH* Schleswig–Holstein, *HH* Hamburg, *BB* Brandenburg, *MV* Mecklenburg-West Pomerania, *TH* Thuringia, *ST* Saxony-Anhalt, *SL* Saarland, *HB* Bremen

The regional distribution of changes in capacities and, therefore, coverage per inhabitants showed a north–south gradient (Fig. 2). While we observed an increase or no change in CPU coverage in southern states like Bavaria, Rhineland-Palatinate, Hesse, Saxony, Thuringia and Saarland (except for Baden-Wuerttemberg), we saw a decrease in CPU coverage in North Rhine-Westphalia, Lower Saxony, Saxony-Anhalt, Brandenburg, Mecklenburg West-Pomerania (except for Schleswig–Holstein) and in the city states of Berlin, Hamburg and Bremen.

### Changes in reorganizational characterisation

**Fig. 3:**
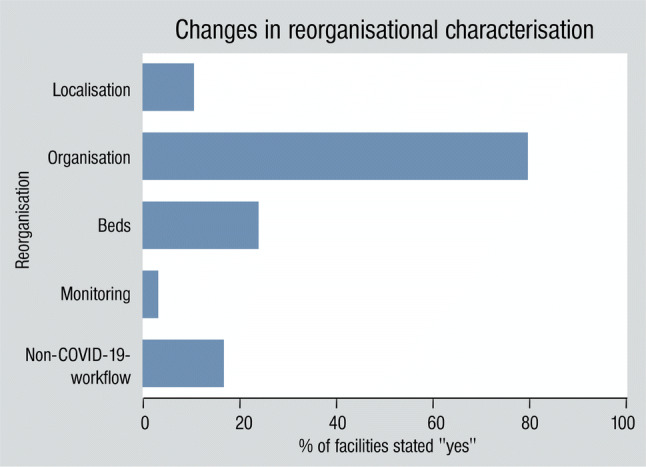
Changes in reorganizational characterisation. We observed changes in localisation, organisation, beds, monitoring and non-COVID-19-workflow

25 CPUs (10%) were affected by changes in location, 206 (81%) reported further changes in organisation, 59 (23%) reported changes in bed capacity or equipment, 7 (3%) reported changes in monitoring and 40 (19%) reported that the regional pandemic plans affected processes in non-SARS-CoV-2-infected patients (Fig. 3).

### Administrational responsibility for reorganisation during COVID-19 pandemic

The vast majority of hospitals (83%) created a crisis management/taskforce for decisions concerning (re-)organization during pandemic outbreak. In 7%, the hospital or hospital group management was responsible; in a few cases (2%), the medical director was reported responsible. No (re-)organisation was primarily carried out by the health department as the political organ. Of the eight CPUs that closed their admittance, the decision was led by crisis management/taskforce (five CPUs), hospital management (two CPUs) and the medical director (one CPU), offering bail-out strategies with transfer to neighbouring CPUs in four cases (50%).

### Admittance of patients with acute chest pain and suspected COVID-19

25% of the hospitals would have admitted chest pain patients with risk for COVID-19 infection to their CPU under isolation conditions, regardless of the internal CPU location. 28% of the hospitals would have admitted patients to the ER under isolation conditions, 19% directly to the ICU, 9% to the IMC unit. 12% would have admitted patients to newly created isolation COVID wards. Others included admissions to isolation containers/tents (< 1%) and other hospitals (2%). 3% (7 in total) of the hospitals were not able to provide standardised monitoring for chest pain patients with suspected SARS-CoV-2 infection.

### Capability of mechanical ventilation in CPU

In the pre-pandemic phase, 20% of CPUs reported capability for mechanical ventilation (51 beds). With many hospitals increasing their capacities for mechanical ventilation, in the pandemic phase, one-third of the CPUs were capable of mechanical ventilation (87 beds). We found the highest total increase of 12 beds in the state of North Rhine-Westphalia.

## Discussion

Cardiovascular disease patients are at particularly high risk by COVID-19, either directly due to frailty, susceptibility, SARS-CoV-2-related myocardial injury and medication or indirectly because of negative implications resulting from medical system changes [12–15]. The latter may bear the risk of postponing possibly life-saving procedures or vice versa to produce additional patients with acute coronary syndromes and late referral, additionally stressed by reduced access to medical care because of fear for contagion [16]. Resulting from the pandemic outbreak of the novel SARS-CoV-2, the caregivers worldwide pass through a process of reorganisation and restructuration trying to maintain adequate coverage for chest pain patients. Due to limited resources especially in regional hospitals, but also due to limited medical personnel due to infection or quarantine, given diagnostic and even therapeutic standard operating procedures (SOPs) had to be adapted and/or reorganised leading to potential shortening of diagnostic protocols as well as pre- and in-hospital delays [16–18]. As such, German CPUs as part of the German emergency structure were facing a new and unknown situation with no former experience or planned coping strategies. Very early in this process, the GCS took position and addressed the need to maintain infrastructure for handling patients with acute cardiac symptoms [4, 5, 19].

In contrast to 2016 findings, where the nationwide German CPU network—including 230 certified CPUs at this time—provided a mean of 1 CPU bed per 65.000 inhabitants, we found a pre-pandemic versus pandemic CPU bed capacity of 1 CPU bed per 47.700 inhabitants versus 1 CPU bed per 49.000 inhabitants, respectively [9]. While university hospitals were able to increase the total CPU beds, academic teaching hospitals and other health care providers had to reduce CPU beds. Nonetheless, the type of facility did not automatically predict structural resources. As a consequence of restructuration of the first hospital medical encounter location, e.g. enlargement of ER bed capacities or creation of alternative pathways for infectious patients, we saw a relocation of CPU beds out of the ER into separate wards. In some individual cases, this led to a total lack of standardised monitoring. Interestingly, we observed no reduction in CPU beds when being located next ICU beds. This may be due to a massive increase in overall ICU bed capacity or by the low admittance of ICU COVID-19 patients so far and thus may change in times with higher patient volume [20]. Some IMC unit beds may have been upgraded to ICU beds, which may explain at least in part the decrease in CPU beds in IMC units. The observed expanded capacity of mechanical ventilation could be explained as possible preparation for upgrading CPUs in case of rededication to a ventilation ward. We primarily hypothesised that the burden of infection cases per federal state will be reflected in the number of CPU beds per inhabitants. Nevertheless, the observed north–south gradient could neither be explained by the number of infection cases nor by population demographics. With the growing need in ICU beds for COVID-19 patients, we see a possible competition for the 25 CPUs integrated into ICUs, as relocations of beds may become necessary in a short time frame. Reorganisation also bears implications on CPU-specific SOPs. As to the pandemic-adapted plans, about one-fourth of patients presenting with acute chest pain and suspected SARS-CoV-2 infection would have been admitted to pre-existing separate CPUs, where existing SOPs are established and trained. With the restructuration of first hospital medical encounter location and ERs being the place to go for many patients, one-third of the patients would have been admitted to the ER, where CPU processes were implemented, although dedicated CPUs are likely to provide certain superiority with respect to diagnostics and treatment [21].

So far and with all necessary methodical caution, we see a comparably low fatality rate of 1.2% across Germany (due date: April 1th 2020) [22]. We do not anticipate that maintaining high CPU capacities directly affected this rate. However, the lower rate of patients with acute chest pain being admitted and, therefore, treated raises deep concerns towards an evolving substantial increase in early and late infarct-related morbidity and mortality. As this phenomenon is partly explained by system-related factors such as increased critical time intervals or reduced diagnostics, offering enough capacity for cardiac patients at risk will still contribute to reduce collateral fatality rates [16, 23, 24]. Further studies should be performed in the transition and inter-pandemic phase to elucidate COVID-19-associated cardiovascular morbidity and mortality as well as COVID-19-associated morbidity and mortality of non-COVID-19 patients due to structural changes in hospitals and possible delays in first medical contact and emergency medical services. To maintain a high-standard care for cardiovascular patients and especially those with acute ischemia or critical cardiac condition, development of inter- and intra-hospital clinical guidelines on managing acute cardiovascular diseases to support medical care providers during COVID-19 pandemic is highly recommended [18].

### Study limitations

Only certified CPUs were included in the study; of those 9% were not reached or refused to take part in the survey. The analysis represents a descriptive observational study without further statistical exploration. The fact that the structured telephone interview was conducted from March 18th to April 1st 2020 bears the risk of bias as we were faced with a highly dynamic situation even within those 15 days and a change of strategy may have been evolved in some hospitals even within this short period of time. However, the order of phone calls was performed by the date of initial certification rather than by location so that the bias is thought to be stretched throughout all regions/federal states. Federal state-specific percentual changes are dependent on the total amount of CPUs reached within the state and should, therefore, viewed with care.

## Conclusion

Germany has a strong and tight network of CPUs. With the present survey, we can demonstrate that the CPU bed capacity was held constant despite all reorganisation pressure during the COVID-19 pandemic allowing us to maintain adequate coverage for all chest pain patients. CPUs organised in a separate setting within the department of cardiology seems to be in favour. Providing uniform, central and binding guidelines on managing chest pain patients in SARS-CoV-2-infected and non-infected patients to unify care may be profitable, at least each CPU should be advised to develop corresponding clinical SOPs. Such system may be transferred to a European level, which has recently been put into action [25, 26]. To implement obligatory partaking in central data collection to ensure nationwide quality control could bear further benefit.

## Data Availability

The data underlying this article will be shared anonymised on reasonable request to the corresponding author.
